# Older hip fracture patients: three groups with different needs

**DOI:** 10.1186/1471-2318-10-65

**Published:** 2010-09-18

**Authors:** Anette H Ranhoff, Kristin Holvik, Mette I Martinsen, Kirsti Domaas, Ludvig F Solheim

**Affiliations:** 1 Department of Medicine, Diakonhjemmet Hospital, PB 23 Vinderen, 0319 Oslo, Norway; 2 Department of Surgery, Diakonhjemmet Hospital, Oslo, Norway

## Abstract

**Background:**

Norway, and particularly Oslo, has the highest reported incidence of hip fractures in the world. It is increasingly common to care for older hip fracture patients in orthogeriatric units where orthopaedic care is combined with interdisciplinary geriatric care. The characteristics and needs of older hip fracture patients are poorly described. The aim of this paper is to describe the characteristics of these patients in order to better understand their need for care and rehabilitation.

**Methods:**

This is an observational study based on a quality register for all patients 65+ years in an orthogeriatric unit who are operated for a hip fracture. The unit covers 250,000 inhabitants in Oslo. Patient data were collected in the aim of quality control. The quality database includes demographic, medical, and functional data collected from routine assessment by the interdisciplinary team.

**Results:**

From January 2007 to September 2009, 1010 patients, included 241 (24%) from long-term care institutions, were enrolled in the database. Mean age was 85.1 years (SD 7.1), 76% were female, and 83% had experienced an indoor fall. Chronic diseases were registered in 88%, and 38% of the community-dwelling patients had pre-fracture cognitive impairment defined as IQCODE-SF > 3.6. Complications were observed in 51% of the patients, of which the most common were a need for blood transfusion, delirium, and urinary tract infections. Post-operative orthopaedic infections were rare (3.1%). Patients from long-term care were older, (87 vs. 84 years, p < 0.001), more had American Society of Anaesthesiologists (ASA) score >/= 3 (67% vs. 48%, p < 0.001) and a higher number of chronic medical conditions (mean 2.2 vs. 1.6, p < 0.001). Among community-dwelling patients, those who had fallen indoors were older, more often female, had ASA score >/= 3, chronic medical conditions, impairment in pre-fracture ADL and cognitive function, and more complications during hospital stay.

**Conclusions:**

Older hip fracture patients in this orthogeriatric unit may be divided into three groups; patients who are relatively fit and have experienced outdoors falls (17%), frail community-dwelling patients who have fallen indoors (59%), and patients from long-term care institutions (24%). Different caring pathways are needed for these groups.

## Background

Older patients with hip fractures represent an important and large patient group in the acute hospitals. Norway, and Oslo in particular, has the highest reported incidence of hip fractures in the world [1]. In a Government report concerning acute medical care in Norway [2] hip fracture is listed as the most common cause of admission for acute hospital care among persons 90 years and older.

There are strong arguments for improvement of osteoporosis treatment and fall prevention, both in order to reduce future falls and fracture risk, as well as to improve survival and functional outcome. As these patients are known to be older, have comorbid diseases and to be dependent in activities of daily living (ADL), there is a demand for special care units with interdisciplinary geriatric care integrated with orthopaedic care [3-6]. Scientific evidence for orthogeriatric care is described in guidelines from the British Orthopaedic Association (http://www.boa.ac.uk).

Most studies describing patients with hip fractures have excluded patients admitted from long-term care and patients who did not provide informed consent. Within the population of hip fracture patients there may be large heterogeneity in characteristics and needs. Patients from long-term care may have specific characteristics, and among community-dwelling patients, falling outdoors may be a marker of a higher functional level in activities of daily living (ADL). The aim of this paper was to describe the characteristics of older hip fracture patients admitted to an orthogeriatric unit, and to compare patients from long-term care with community-dwelling patients with outdoor and indoor falls, in order to better understand their need for care and rehabilitation.

## Methods

This was an observational study based on routine data that was de-identified and entered into a database in the aim of quality improvement in an orthogeriatric unit for patients 65 years and older with hip fractures. The unit covers a population of approximately 250,000 inhabitants, half of the population of Oslo. It is organised in Department of Surgery, and it has 20 ordinary beds and 4 observation beds for pre- and postoperative care. Orthopaedic surgeons, a geriatrician, specially trained nurses, occupational therapist and physiotherapists are working in the unit. The organisation and principles of care of the unit are described in table 1.

**Table 1:** Organisation, routines, staffing and resources in the orthogeriatric unit

Unit size and location	20 beds (3 single rooms and 7 double rooms, 1 three-person room) 4 observation beds for pre- and postoperative care
Staff	Nurse factor 1.65 2 physiotherapists 0.8 occupational therapist (specialized in geriatrics) 0.2 clinical nutritionist
Training and education	Two weeks training for all nursing staff before work-start. Weekly teaching-lessons of 45 min for all staff.
Interdisciplinary team	Nurse, nurse-assistant, physiotherapist (PT), occupational therapist (OT), clinical nutritionist, pharmacist, orthopaedic surgeon and geriatrician.
Comprehensive geriatric assessment	Assessment of pre-fracture ADL (Barthel Index) and cognitive function (IQCODE-SF) by interview with next-of-kin (OT or nurse). ADL assessment 1.-3. day after surgery and at discharge. MMSE at discharge (OT). An integrated care plan including rehabilitation and discharge planning approved by the interdisciplinary team meeting (twice weekly).
Prevention of complications	Systematic prevention of complications: delirium, falls, tromboembolism, nosocomial infections, pressure sores and wound infections. Screening for urinary tract infections. Removal of bladder catheter within 24 hours after surgery. Screening for urinary retention by bladder scans. Prevention of constipation. Blood transfusion at haemoglobin less than 8 mg/l, at higher levels in patients with coronary artery disease. Systematic pain control (by protocol).
Nutrition	Oral liquid supplements to all patients up to two hours before surgery. Fortified diet; small energy-dense portions Measurement of weight and height, calculation of BMI. Patients with BMI < 22 are assessed by a clinical nutritionist. Additional nutritional supplements to patients with low BMI.
Rehabilitation	Mobilisation on day one after surgery. Daily ADL-training and walk training with help from nursing staff. Counselling and training with PT and OT. Patients are emphasized to be as independent as possible, to leave their rooms and to take all meals in the dining room. Follow up by PT at three months.
Prevention of subsequent fractures	Fall assessment and multifactor intervention to prevent new falls. Bone mineral density measurement and assessment for osteoporosis treatment (calcium, vitamin D and bisphosphonates).

### Data collection

We have used data on patients admitted to the orthogeriatric unit from January 2007 to September 2009. Patients younger than 65 years, those with other diagnoses than proximal femoral fracture (S72.0-S72.2 according to ICD-10) and those who stayed in other wards than the actual orthogeriatric unit were excluded. All data were collected during routine care and a registration form was completed during the interdisciplinary ward meetings. Data on these forms that fulfilled the inclusion criteria were de-identified and transferred to a database. Demographic data and data about fracture type, type of surgery and comorbidity were collected from all patients. Data about activities of daily living (ADL) and cognitive function were collected from community-dwelling patients only, as nursing home residents are considered to need help with most ADLs and more than 80% of them suffer from dementia [7]. Severity of co-morbidity expressed by American Society of Anaesthesiologists (ASA) score [8] was registered by anaesthesiologists. The score ranges from I (healthy) to V (moribund). Type of fracture, surgical procedure and time of surgery was registered by orthopaedic surgeons, while the geriatrician registered medical co-morbidities and complications during stay in hospital.

The occupational therapist, physiotherapists and nurses collected data about function. Pre-fracture cognitive function was assessed by an interview with a next-of-kin using the short form of the Informant Questionnaire on Cognitive Decline in the Elderly (IQCODE-SF) scoring from 0 to 5. A score of > 3.6 indicate cognitive impairment [9,10]. The Barthel ADL Index (BI) was used to assess pre-fracture function (interview with patient and next-of-kin), and function at discharge (observation by staff). BI is scored from 0 (totally dependent) to 20. A score of 19 or 20 indicates independency in daily life. Nurses measured body weight and height and calculated body mass index (BMI). Delirium was detected by using the Confusion Assessment Method (CAM) [11]. Mortality, length of stay (LOS) and place of discharge were recorded at discharge.

### Statistics

Data were analysed using SPSS version 14.0. Normally distributed continuous variables are presented as mean and standard deviation (SD), while non-normally distributed variables are presented as median with 25 and 75 percentile (Barthel Index, MMSE, LOS, waiting time for surgery). Stratified analyses were performed to compare community-dwelling patients who had fallen indoors and outdoors, and patients with IQCODE-SF > 3.6 and the others. Continuous normally distributed variables (age and BMI) were compared using t-test. Continuous non-normally distributed variables (Barthel Index and MMSE) were compared using the Mann-Whitney U test. Percentages were compared by Chi square test. For the variables BMI, Barthel Index and IQCODE-SF, patients with missing data were excluded from the analyses.

### Ethics

This is an observational study based on routine data and no experimental intervention is performed. All data are de-identified when transferred into the database. The patients are not asked to give informed consent. The Norwegian Social Science Data Services (NSD) has approved the database.

## Results

A total of 1010 patients, 65 years and older, were admitted to the orthogeriatric unit and underwent hip fracture repair from January 2007 to September 2009. Characteristics of the patients are shown in table 2. Mean age was 85.0 years and the oldest patient was 105 years old. Three of four (76%) were female and 83% had fallen indoors. The most common types of fracture were fracture of neck of femur (55%) and pertrochanteric fracture (39%). Totally 34% received a hemiarthroplasty.

**Table 2:** Characteristics of hip fracture patients 65 years and older admitted to an orthogeriatric unit January 2007 - September 2009 (n = 1010)

Number (%) if not otherwise stated	
	
Age, years (mean (SD))	85.0 (7.1)
Gender, women	763 (75.5)
Nursing home residents	234 (23.2)
Indoor fall	838 (83.0)
American Society of Anesthesiologists (ASA) score, n = 1009:	
I - Healthy	10 (1.0)
II - Mild systemic disease	468 (46.4)
III - Severe systemic disease	505 (50.0)
IV - Incapacitating/life-threatening systemic disease	26 (2.6)
V - Moribund	0
Type of fracture	
- Fractur neck of femur	558 (55.2)
- Pertrochanteric fracture	391 (38.7)
- Subtrochanteric fracture	61 (6.0)
Type of surgery	
- Screws	191 (18.9)
- Hemiarthroplasty	343 (34.0)
- Dynamic Hip Screw	357 (35.3)
- Intramedullary Hip Screw	115 (11.4)
- Other (THR or girdlestone)	4 (0.4)
Body mass index, kg/m2, mean (SD) (n = 550)	22.6 (3.8)
Length of stay, days, median (25, 75 percentile)	10 (4, 16)
Waiting time for surgery, hours, median (25, 75 percentile) (n = 1005)*	11 (5, 19)
Place of discharge	
- Short-term stay in nursing home for rehabilitation and evaluation	336 (33.3)
- Long-term care institution	234 (23.2)
- Rehabilitation centre	194 (19.2)
- Home	176 (17.4)
- Other hospital ward	45 (4.5)
Died during hospital stay	25 (2.5)

Almost one-fourth (24%) were admitted from long-term care institutions, and, except for two, these patients had fallen indoors. They were older (87.1 vs. 84.3 years, p < 0.001), more had ASA score >/= 3 (67% vs. 48%, p < 0.001) and they had a higher number of chronic medical conditions (mean 2.2 vs. 1.6, p < 0.001).

**Figure 1:**
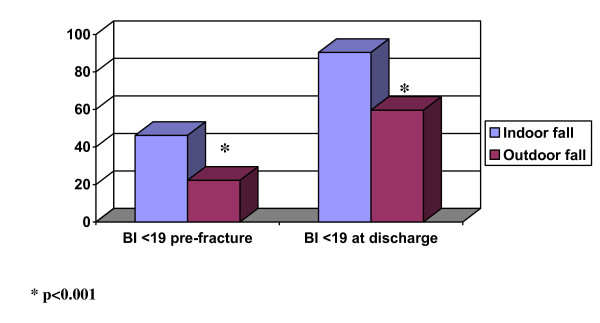
**Proportion of patients (%) with ADL function (Barthel Index) < 19 pre-fracture and at discharge from hospital according to indoor or outdoor fall**.

Community-dwelling patients with indoor and outdoor falls are compared in table 3. Patients who had fallen indoors were older, more were women, they had poorer general health (ASA score), more chronic medical conditions, impairment in pre-fracture ADL (BI) and cognitive function, and more complications during hospital stay. At discharge, 90% of patients with indoor falls were dependent in ADL (BI < 19) vs. 60% of those with outdoor falls (p < 0.001); see figure 1.

**Table 3:** Characteristics of community-dwelling patients.

Characteristic	All community-dwelling (n = 769)	Community-dwelling who fell outdoors (n = 170)	Community-dwelling who fell indoors (n = 599)	p	
Age, years, mean (range)	84.3 (65-100)	82.7 (66-100)	84.8 (65-100)	0.001	
Gender, n (%) female	584 (75.9)	110 (64.7)	474 (79.1)	< 0.001	
ASA score, n (%) ≥3	368 (47.9)	52 (30.6)	316 (52.8)	< 0.001	
BMI, n (%) < 20 kg/m^2 ^(n = 520) ^1^	129 (24.8)	24 (19.4)	105(26.5)	0.11	
Barthel Index pre-fracture < 19, n (%) (n = 493)^2^	203 (41.2)	23 (22.3)	180 (46.2)	< 0.001	
Barthel Index at discharge < 19, n (%) (n = 316)^3^	265 (83.9)	40 (59.7)	225 (90.4)	< 0.001	
IQCODE-SF > 3.6, n (%) (n = 511)^4^	192 (37.6)	22 (20.0)	170 (42.4)	< 0.001	
**Chronic medical disorders (from patient's records), n (%)**					
Dementia	160 (20.8)	16 (9.4)	144 (24.0)	< 0.001	
Pulmonary disease	107 (13.9)	17 (10.0)	90 (15.0)	0.10	
Major vision impairment	81 (10.5)	12 (7.1)	69 (11.5)	0.09	
Major hearing impairment	73 (9.5)	15 (8.8)	58 (9.7)	0.73	
Musculoskeletal disorder	70 (9.1)	20 (11.8)	50 (8.3)	0.17	
Endocrine disorder (other than diabetes)	67 (8.7)	15 (8.8)	52 (8.7)	0.95	
Diabetes mellitus	60 (7.8)	9 (5.3)	51 (8.5)	0.17	
Cerebrovascular disease	56 (7.3)	8 (4.7)	48 (8.0)	0.14	
Psychiatric disorder	54 (7.0)	6 (3.5)	48 (8.0)	0.043	
Osteoporosis with previous fracture	51 (6.6)	7 (4.1)	44 (7.3)	0.14	
Neurologic disorder	44 (5.7)	8 (4.7)	36 (6.0)	0.52	
Cancer	42 (5.5)	9 (5.3)	33 (5.5)	0.91	
Renal failure	22 (2.9)	5 (2.9)	17 (2.8)	0.94	
**Type of medical complication observed during the stay, n (%)**					
Need for blood transfusion	207 (26.9)	34 (20.0)	173 (28.9)	0.021	
Delirium (positive CAM)	169 (22.0)	31 (18.2)	138 (23.0)	0.18	
Urinary tract infection	161 (20.9)	19 (11.2)	142 (23.7)	< 0.001	
Pneumonia	88 (11.4)	12 (7.1)	76 (12.7)	0.042	
Fall	59 (7.7)	12 (7.1)	47 (7.8)	0.73	
Cardiac complications (myocardial infarction, hearth failure, atrial fibrillation)	49 (6.4)	11 (6.5)	38 (6.3)	0.95	
Postoperative wound infection	26 (3.4)	6 (3.5)	20 (3.3)	0.90	
Haematoma	17 (2.2)	2 (1.2)	15 (2.5)	0.39	
Bedsore	11 (1.4)	1 (0.6)	10 (1.7)	0.47	

Outdoor and indoor falls

^1 ^BMI was registered in 124 outdoor-fallers and 396 indoor-fallers

^2 ^Barthel Index pre-fracture was registered in 103 outdoor-fallers and 390 indoor-fallers

^3 ^Barthel Index at discharge was registered in 67 outdoor-fallers and 225 indoor-fallers

^4 ^IQCODE-SF was registered in 110 outdoor-fallers and 401 indoor-fallers

IQCODE-SF score was registered from 511 (66%) of the community-dwelling patients. There was no difference in age, gender, or ASA score between those registered and those not. Pre-fracture cognitive impairment (IQCODE-SF > 3.6) was found in 192 (38%) patients. The patients who had cognitive impairment were older (mean 86.1 versus 83.3 years, p < 0.001), more had ASA score >/= 3 (55% versus 43%, p = 0.005) and more had pre-fracture impairment in ADL (BI < 19) (66% versus 26%, p < 0.001). Those with cognitive impairment had also more chronic medical conditions (mean 2.1 versus 1.5, p < 0.001) and complications (mean 1.0 versus 1.5, p < 0.001).

BMI was registered in 521 (68%) of the community-dwelling patients. Those registered with BMI tended to be younger (mean 84.0 versus 85.0 years, p = 0.07) and fewer had ASA score >/= 3 (46% versus 53%, p = 0.047). There was no significant difference in prevalence of low body weight (BMI < 20 kg/m^2^) between patients with or without cognitive impairment or between patients who had fallen indoors or outdoors.

## Discussion

The majority of our patients were very old, were women, and had chronic medical conditions. Low body weight, cognitive impairment and impaired pre-fracture ADL were common. At discharge, 84% of the community-dwelling patients had BI < 19, indicating a need for help in daily life and a need for continuation of a rehabilitation program to restore pre-fracture function.

Based on the results we may divide the patients into three groups with increasingly failing health and function: Community-dwelling elderly who have fallen outdoors (17%), community-dwelling elderly who have fallen indoors (59%) and patients from long-term care institutions (24%). These findings harmonize well with a study from the UK where different risk profiles for outdoor and indoor falls in older home-dwelling people were found. Indoor falls were associated with frailty and excess mortality, while outdoor falls were associated with compromised health status in more active people [12]. The three groups have different characteristics and needs, but there are some overlapping problems between them. Low body weight is a common trait in all the hip fracture patients, which is equally distributed between patients from the community and is also believed to be common among patients from long-term care institutions [13], although we do not have sufficient BMI data to support this. We found pre-fracture cognitive impairment among 20% of the patients who had fallen outdoors, and in 38% of all community-dwelling patients. In the patient records only 21% were registered with dementia, demonstrating the importance of using systematic assessment with IQCODE-SF to detect cognitive impairment probably due to dementia [10]. Among patients admitted from long-term care facilities, the prevalence of cognitive impairment is probably much higher, as approximately 80% of patients in Norwegian long-term care institutions have dementia [7].

The strength of this study is that we have data also from patients that are very old, frail and suffering from dementia, and who are often excluded from similar studies. However, unfortunately, the data are not complete for all patients. Missing data are mostly attributed to time-consuming assessments (IQCODE-SF, Barthel Index and BMI), which may have a low priority on a busy ward. Although age, gender, and ASA score did not differ between patients who had been assessed with IQCODE-SF and Barthel Index, and those who had not, there may be some bias. We have BMI data for only 68% of the community-dwelling patients and few of the long-term care patients, indicating an area for quality improvement in the unit. Those registered with BMI tended to be younger and were healthier, probably explaining that they were easier to get on a scale.

Delirium was registered in 22% of the community-dwelling patients, while other studies have reported more than 50% during hospital stay [14-17]. In our study, delirium was registered mainly post-operatively and after the patient had moved out from the recovery unit. Nevertheless, delirium is a major problem in older hip fracture patients and systematic prevention has proved to be effective [14-16].

Most of these patients are believed to benefit from orthogeriatric care including optimal medical care, fall prevention assessment, and rehabilitation in order to restore pre-fracture functional status [3-6]. However, the three groups of patients have different needs and may benefit from different care pathways. The community-dwelling patients who have fallen outdoors are the most healthy and youngest. Assessment of osteoporosis and evaluation for treatment with bisphosphonates in addition to calcium and vitamin D supplementation is particularly important in this group [18]. The more frail patients who have fallen indoors may be most in danger for institutionalisation [12] and may benefit from careful discharge planning and continuation of the rehabilitation program after discharge. For patients from long-term care institutions, hip protectors [19] and proper nursing care are probably more important than a multifactor fall prevention program and intensive rehabilitation.

Rehabilitation of patients with dementia is challenging, but not impossible. Patients with mild and moderate dementia in Finland benefited from systematic rehabilitation after hip fracture, and the majority could be discharged to their own home [20]. In a study comparing intensive muscle strength exercise with ordinary rehabilitation, patients with dementia improved their function most [21].

An alternative way of selecting patients to different care plans could be to use this information and select patients with and without cognitive impairment to different care plans. In total, about 50% of our hip fracture patients have cognitive impairment, and most of them dementia.

A combination of good orthopaedic care and comprehensive geriatric care seems to be a practical way to care for patients such as those described in this study [22,23]. However, the effect and appropriateness of more specific care plans for different groups of patients should be studied further.

## Conclusions

Hip fracture patients in this orthogeriatric unit have high age, are frail with comorbid diseases, and many have cognitive impairment. They may be divided into three groups; patients who are relatively fit and have experienced outdoor falls, frail community-dwelling patients who have fallen indoors, and patients from long-term care institutions. Different caring pathways are needed for these groups.

## Competing interests

The authors declare that they have no competing interests.

## Authors' contributions

AHR was responsible for conception and design of the study, acquisition of medical data (comorbidity, complications), analysis and interpretation of data and drafting of the manuscript. KH participated in conception and design, acquisition, analyses and interpretation of data. MIM participated in conception and design, acquisition of administrative and functional data, analyses and interpretation of data. KD participated in conception and design, practical procedures and acquisition of functional data. LFS participated in conception and design, acquisition of surgical data (type of fracture and operation method), analysis and interpretation of data and drafting of the manuscript.

All authors have read and approved the final manuscript.

## Authors' information

AHR is a geriatrician, PhD and professor of geriatrics. KH is a clinical nutritionist and epidemiologist, PhD. MIM is a registered nurse. KD is an occupational therapist. LFS is an orthopedic surgon, PhD and the chair of the department.

## Acknowledgements

The authors would like to thank all the patients and the staff at the Orthogeriatric unit in Diakonhjemmet Hospital. The study is entirely sponsored by Diakonhjemmet Hospital.

## Pre-publication history

The pre-publication history for this paper can be accessed here:

http://www.biomedcentral.com/1471-2318/10/65/prepub
